# Assessing the feasibility and acceptability of a cluster-randomized study of cognitive behavioral therapy for chronic insomnia in a primary care setting

**DOI:** 10.1186/s12875-021-01429-5

**Published:** 2021-04-16

**Authors:** Isabel Torrens, Magdalena Esteva, Caterina Vicens, María Rosa Pizá-Portell, María Clara Vidal-Thomàs, Cristina Vidal-Ribas, Patricia Lorente-Montalvo, Elena Torres-Solera

**Affiliations:** 1 Santa Ponsa Health Center, Majorca Department of Primary Care, Baleares Health Service [IbSalut], Riu Síl, 25, 07180 Santa Ponça, Majorca Spain; 2 Balearic Islands Health Research Institute (IdISBa), Edificio S, University Hospital Son Espases, Carretera de Valldemossa, 79, 07120 Palma, Majorca Spain; 3 Primary Care Research Unit, Majorca Department of Primary Care, Baleares Health Service [IbSalut], Escola Graduada 3, 07001 Palma, Spain; 4 Preventive Activities and Health Promotion Research Network (REDIAPP), Barcelona, Spain; 5 Son Serra Health Center, Majorca Department of Primary Care, Baleares Health Service [IbSalut], Carrer de Matamusinos, 22, 07013 Palma, Majorca Spain

**Keywords:** Insomnia, Treatment, Primary care, Cognitive behavioral therapy, Randomized controlled trial, Feasibility study, Pilot study

## Abstract

**Background:**

Cognitive behavioral therapy for chronic insomnia (CBT-i) is the treatment of choice for this condition but is underutilized in patients who attend primary care. The purpose of the present feasibility-pilot study was to assess the feasibility and acceptability of a cluster-randomized study of CBT-i in a primary care setting.

**Methods:**

This study, performed at two primary health care centers in Majorca, Spain, was a mixed methods feasibility-pilot study of a parallel cluster-randomized design comparing CBT-i and usual care (UC). Patients were included if they were 18 to 65 years-old; had diagnoses of chronic insomnia according to the Insomnia Severity Index (ISI ≥ 8); had insomnia for more than 3 months. Twenty-five GPs and nurses and 32 patients were randomly allocated to two groups. The main outcome of the intervention was improvement of dimensions of sleep quality, measured using the Spanish version of the Pittsburgh Sleep Quality Index, at baseline and at 3 months after the intervention. Other primary outcomes of the study were the feasibility and applicability of the intervention, collected through nominal groups. A thematic analysis was performed to classify primary care provider (PCP) proposals. Additionally, we assessed the recruitment process, compliance with the intervention sessions, and patient retention.

**Results:**

We adapted the CBT-i approach of Morin to a primary care context. After intervention training, PCPs expressed the need for more extensive training in the different aspects of the therapy and the discussion of more cases. PCPs considered the intervention as adequate but wanted fewer but longer sessions as well as to discard the cognitive restructuring component. PCPs considered it crucial to prepare each session in advance and to establish a specific agenda for the CBT-i. Regular reminders given to PCPs and patients were suggested to improve study participation. Compared to the UC group, higher proportions of patients in the intervention group had short sleep latency, slept for longer than 5 h, and had fewer sleep disruptions.

**Conclusions:**

This feasibility-pilot study identified several key issues that must be addressed before performing a CBT-i intervention in future clinical trial in a primary care setting.

**Trial registration:**

NCT04565223. (Clinical trials.gov) Registered 1 September 2020—Retrospectively registered.

## Background

Insomnia is a disorder characterized by difficulties in falling asleep and staying asleep during the night or the perception of having a non-restful sleep [1]. It is the most common sleep disorder in the general population, affecting 6.4% of people in Spain. Moreover, 20.8% of individuals in Spain experience symptoms of insomnia at least 2 or 3 times per week [2, 3]. Even higher percentages have been reported for other European countries [4–7]. Chronic insomnia is a significant burden to society because it can lead to the development of physical [8, 9] and psychological disorders, increase the risk of road accidents [10, 11], and lead to excessive sick leave [9] and greater use of health care services [12, 13].

In spite of its high prevalence and association with multiple health problems, there is only limited availability of effective treatments for insomnia. Hypnotic medications (i.e., benzodiazepines and/or Z drugs) often effectively alleviate symptoms, but can lead to side-effects, dependency, and tolerance [14, 15]. Cognitive behavioral therapy for insomnia (CBT-i) is a multicomponent intervention which focuses on cognitive and behavioral factors that contribute to sleep disorders [16]. Recent systematic reviews showed that CBT-i provided by psychologists and psychiatrists is more effective than usual treatments [17] and medications, and also lasts longer [18], even in patients with comorbidities [19].

However, the efficacy of CBT-i when applied by general practitioners (GPs) or primary care (PC) nurses has not been sufficiently demonstrated. A study that compared CBT-i provided by PC nurses with usual care provided by a GP indicated that CBT-i was associated with improvements in self-reported sleep latency, wakefulness after sleep onset, and sleep efficiency, and that patients receiving usual care did not improve [20]. Another study of CBT-i provided by PC nurses [21] also showed that this intervention provided distinct reductions in night awakening and sleep latency.

CBT-i is underutilized by GPs and PC nurses for several reasons. These include the following: (1) CBT-i is not designed for use by primary care providers (PCPs) [22], (2) it requires significant effort to motivate patients to change their behaviors [23], (3) it can be very time-consuming; (4) PCPs typically lack the skills to administer CBT-i; and (5) many PCPs do not consider insomnia to be an important issue [24, 25]. However, most patients with insomnia are diagnosed in PC settings [26]. Thus it is necessary to have an effective CBT-i that is adapted to the skills of PCPs, because implementation of therapy in this setting may have a greater impact.

In the present work, we performed a mixture of a feasibility study and a pilot study of the effectiveness of a CBT-i intervention in PC patients with insomnia. This study examined many of the procedures to be used in a future trial in an effort to prevent problems during this upcoming trial [27].

### Objectives

The primary objectives of this feasibility-pilot study were:

1. To design and adapt a brief CBT-i intervention to be provided by PCPs for the treatment of chronic insomnia in individuals who are 18 to 65 years-old.
2. To define usual care (UC) for the treatment of chronic insomnia provided by PCPs as a comparative intervention (control group).
3. To assess the training activities for the CBT-i intervention by determining GPs’ and nurses’ satisfaction with the content and applicability of the intervention.
4. To explore the acceptability of the intervention to GPs and nurses.
5. To assess PCPs and patient recruitment, follow-up, and adherence to the intervention.

The secondary objective was to assess the quality of sleep in patients after 3 months of the CBT-i.

## Methods

### Design

This was a mixed-methods feasibility-pilot study of a two-arm cluster-randomized trial comparing CBT-i and usual care (UC). The allocated groups were PC doctors and nurses (the clusters). The study was performed from September 2014 to April 2015 at two primary health care centers of Majorca (Spain) with 56,000 registered inhabitants. This study adheres to CONSORT guidelines.

### Intervention

#### CBT-i Intervention

The CBT-i intervention was developed by two family physicians (IT and CV) and two psychologists (ET and MRPP). First, a review of the literature on the use of CBT-i was performed, with a focus on interventions applied in a PC setting. After a literature review, the CBT-i approach designed by Morin [16] was adapted to our setting, in which there were fewer and shorter sessions. The CBT-i included sleep hygiene counseling, stimulus control, cognitive restructuring, relaxation techniques, and benzodiazepine withdrawal (when needed). To conduct the intervention, guidelines for GPs and nurses and graphic and written materials for patients (sleep diary, registry of behavior habits, and cognitive problems) were developed.

#### Description of the intervention

We developed the CBT-i program for implementation by PC family doctors and nurses. The intervention requires active participation of patients for the treatment of insomnia. There were 5 individual sessions of approximately 20 min each, with one session per week or every 2 weeks (based on patient preference) and an additional session for patients undergoing withdrawal of hypnotic medications [28]. Table 1 describes the content of each session.

**Table 1:** Summary of the intervention sessions

	T0	Session 1	Session 2	Session 3	Session 4	Session 5 (hypnotic withdrawal)	Session 5/6	T3
Objectives	1. Baseline evaluation 2. Provide information on the intervention	1. Identify the factors that perpetuate insomnia (behavior-problem) 2. Identify beliefs and predispositions to change 3. Establish objectives (behavior-goal)	1. Review difficulties and changes during the week 2. Identify factors in the patient’s environment related with going to sleep 3. Establish objectives of sleep hygiene modification	1. Review difficulties and changes during the week 2. Identify the times a patient gives to certain habits and develop a timetable 3. Establish objectives of sleep hygiene modification	1. Review difficulties and changes during the week 2. Identify distorting beliefs 3. Transform and implement realistic thinking	1. Review problems related to benzodiazepine consumption 2. Negotiate with the patient to implement a gradual dose reduction	1. Review the Sleep Diary 2. Assess the sessions and identify what has been accomplished 3. Assess what was not accomplished and the next goals	1.Follow up assessment at 3 months after the end of CBT-i
Techniques		Structured interview Relaxation	Sleep hygiene Stimulus Control Relaxation	Restriction of bed time Paradoxical intention Relaxation	Cognitive restructuring Relaxation	Structured interview	Structured interview	
Spreadsheet of tasks		Sleep diary	How are my routines?	How are my times?	How they interfere my thinking’s	To release of written information on gradual reduction of benzodiazepines	Sleep diary	
Duration (min)	20–30	20	20	20	20	20	20	20

This intervention aims to change the habits of the affected patients, and to encourage them to undergo a cognitive and physical deactivation before going to sleep. It aims to help patients to identify and assess how they react before, during, and after sleep and to consider thoughts and behaviors that contribute to insomnia (Behavior-Problem) with the help of a sleep diary. The intervention offers patients a wide range of structured strategies, based on their possible impact (Behavior-Goal). The patient and therapist agreed on the therapeutic objectives before commencement of treatment. These objectives determine the content of the intervention sessions. After each session, patients have a task to work on at home, in which they try to achieve different goals and use different techniques. During treatment, the therapist was available to address doubts and difficulties that the patients may experience. Therapists were asked to record any relevant information from patients that arose during the intervention sessions.

The sleep diary contains a 2-week registry that records the following data: sleeping hours, sleep latency, duration of awakening and number of awakenings, number of times getting out of bed, naps, medication use, and sleep quality. Patients also identified the main difficulties they experienced during the night and reported desired objectives and how they can be achieved.

We developed the CBT-i program for implementation by PC family doctors and nurses. The intervention requires active participation of patients for the treatment of insomnia. There were 5 individual sessions of approximately 20 min each, with one session per week or every 2 weeks (based on patient preference) and an additional session for patients undergoing withdrawal of hypnotic medications [28]. Table 1 describes the content of each session.

This intervention aims to change the habits of the affected patients, and to encourage them to undergo a cognitive and physical deactivation before going to sleep. It aims to help patients to identify and assess how they react before, during, and after sleep and to consider thoughts and behaviors that contribute to insomnia (Behavior-Problem) with the help of a sleep diary. The intervention offers patients a wide range of structured strategies, based on their possible impact (Behavior-Goal). The patient and therapist agreed on the therapeutic objectives before commencement of treatment. These objectives determine the content of the intervention sessions. After each session, patients have a task to work on at home, in which they try to achieve different goals and use different techniques. During treatment, the therapist was available to address doubts and difficulties that the patients may experience. Therapists were asked to record any relevant information from patients that arose during the intervention sessions.

The sleep diary contains a 2-week registry that records the following data: sleeping hours, sleep latency, duration of awakening and number of awakenings, number of times getting out of bed, naps, medication use, and sleep quality. Patients also identified the main difficulties they experienced during the night and reported desired objectives and how they can be achieved.

### Description of usual care

The usual treatment for persistent insomnia in a PC setting was previously described in two cross-sectional studies performed by GPs and family nurses of the Primary Care Majorca Department (Spain) during 2011 and 2015 [29, 30].

The professionals in the control group followed their usual care in treatment of insomnia patients. A previous descriptive study of insomnia treatments used by GPs in Majorca from 2001 to 2012 indicated that more than 95% of them asked patients about habits related to sleep hygiene that might lead to insomnia, 85.1% provided to advice on sleep hygiene measures, 15.1% suggested the use of herbal remedies, and 14.2% suggested CBT-i. More than 33% of GPs prescribed a pharmacological treatment. Benzodiazepines were the most prescribed drugs (33.4%) followed by Z drugs (25.7%). Six of 10 GPs requested a review of the treatment after 1 month of medication use [29].

The usual care provided by nurses was based on a 2014 descriptive study (performed simultaneously with the present study). This study reported that 69.6% of nurses asked patients about their sleeping habits and 48.5% asked about the consequences of their insomnia. A total of 46.4% considered pharmacological interactions and 45.6% gave special consideration to elderly patients. The non-pharmacological treatments they recommended were sleep hygiene measures (76%), herbal remedies (44.9%), and CBT-i (22.4%). About 25% of nurses offered written advice and 81% gave oral advice on sleep hygiene [30].

### Subject recruitment

Sixteen GPs and 7 nurses, who expressed interest in the study, after study presentation in the two health centers, were recruited in September 2014. They were each given a list of 6 patients with chronic insomnia, and each of them selected 2 patients from the list who were eligible. Patients with chronic insomnia who visited their GPs or family nurses could also be recruited. Patients were included if they were 18 to 65 years-old; had diagnoses of chronic insomnia according to the Insomnia Severity Index (ISI ≥ 8) [31, 32] and had insomnia longer than 3 months; and did or did not use a hypnotic medication. Patients were excluded if any of the following conditions were present: secondary insomnia or another sleep disorder, such as restless legs syndrome, parasomnia, or alterations of the circadian rhythm (e.g., due to shift work), or use of a medication that could produce sleep alterations; severe psychiatric disorder; depression (HADS score ≥ 8) or diagnosis of major depression in the clinical records; suicide attempt; use of an antidepressant or anti-psychotic medication; alcohol or drug abuse during the last year; receipt of another CBT-i; chronic disease, such as sleep apnea; diagnosis of dementia or presence of a cognitive deficit (Mini Mental State Evaluation (MME) score < 23); neurodegenerative or oncological disease with poor prognosis; mental or physical incapacities that impeded participation in interviews [33, 34]; acute or chronic pain secondary to a rheumatic disease or another untreated chronic disease; pregnancy; or participation in a previous clinical trial in the participating health centers.

Patients were invited to participate personally or by telephone. If a patient agreed, an appointment in the health care center was scheduled, at which they received oral and written information about the study. If a patient was accepted and deemed eligible, he or she completed the ISI questionnaire to confirm the presence of chronic insomnia, signed an informed consent agreement, and received an appointment for a baseline evaluation.

### Group allocations and blinding

GPs and nurses were randomized (1:1) to the CBT-i intervention group (IG) or the usual care group (UCG) using computer generated random numbers by the Research Unit study manager. PCPs were allocated in the IG after they had selected at least two patients from their list. Two GPs from the research team (CV, and IT) were allocated to the IG because they had the expertise to provide thorough and accurate information about the implementation of the intervention and the need for further training. The two psychologists (ET and MRP-P) delivered the intervention to one patient each in order to share with PCPs which aspects of the intervention could be improved. Blinding of patients and clinicians was not possible due to the nature of the intervention; the assistant researcher was also not blinded, because this person conducted interviews at 3 months that included treatment-specific questions. The objectives of this feasibility-pilot study were focused on assessing clinical trial processes, not on patient outcomes.

### Measurements

#### Feasibility dimensions

The primary measures were: assessment of the acceptability of the training to PCPs (satisfaction with the content, applicability, and ability to provide sufficient training); feasibility of the designed intervention for administration by trained GPs and nurses; and feasibility of the study design (recruitment, follow up, and retention).

### CBT-i training

All doctors and nurses were trained on data collection procedures and administration of questionnaires and psychometric scales. Then, those who were allocated to the intervention group received training in CBT-i from the two psychologists (ET and MRPP). This training (2 sessions, 2 h per session) explained the content of the planned intervention and presented a case report. After the training sessions, a focus group session was conducted in which one investigator (ME), using a script containing questions on the different dimensions, collected the opinions of the doctors and nurses regarding the adequacy, content, and duration of their training, and asked for their ideas about possible improvements.

### Acceptability of the intervention by GPs and nurses

To assess acceptance of the intervention by PCPs, two nominal groups were established, one in each health center. These nominal groups conducted discussions run by one member of the research group (ME) and supported by one observer (MCVT). The group discussions were designed to follow the nominal group process [35] regarding the introduction and clarification of the research task; individual generation of ideas; generation of ideas as a group (proposing ideas for recording on a flip chart); refining the list of ideas by adding, merging, or removing certain ideas; individually ranking the five most important ideas; and then a group review of the aggregate ranking of ideas. The introduction included obtaining consent from all participants. The observer tasks included assuring that all topics in the script were considered during nominal group development. A thematic analysis was undertaken with the aim of identifying the main themes generated during the nominal group’s discussions.

### Pilot study measurements

The variables used to assess the potential effectiveness of the intervention were measured at baseline interview and at 3 months post-treatment; they included the validated Spanish version of the Pittsburgh Sleep Quality Index (PSQI) [36, 37]; the Spanish version of Hospital Anxiety and Depression Scale (HADS) [38, 39]; and use of hypnotic medications. The PSQI is a 19-item self-reported questionnaire that assesses 7 clinical relevant components of sleep quality (subjective sleep quality, sleep latency, sleep duration, sleep efficiency, sleep disturbances, use of sleep medication and daytime dysfunction) in the preceding month. Each component is rated on 0 to 3 point scale referring to the composite score derived from the frequencies of each disturbance, in which 0 corresponds to not in the past month and 3 corresponds to 3 or more times per week, with a global score (the sum of the 7 component scores) ranging from 0 to 21. A cut off score of 5 has been shown to discriminate between good and poor sleepers [36, 37].

The HADS measures anxiety and depression levels. It is a 14-item self-report scale with a 7-item anxiety subscale and a 7-item depression subscale. Each item is scored on a 4-point Likert scale (e.g., 0, as much as I always do; 1, not quite so much; 2, definitely not so much; and 3, not at all), giving maximum subscale scores of 21 for each of depression and anxiety. The questionnaire assesses symptoms over the preceding week. Patients with scores > 10 are considered to have morbidity. Scores between 8 and 10 were considered as borderline cases and scores < 8 were considered to indicate the absence of relevant morbidity [38, 39]. Use of hypnotic medications was also recorded, as reported by patients and verified from clinical records.

In addition, adverse effects at 3 months after the intervention that could be associated with the CBT-i or benzodiazepine discontinuation (tremor, irritability, anxiety, insomnia, and seizure) were recorded, and their severities were rated as none, mild, moderate, or severe. Therapists were asked to report any serious adverse events during the follow-up period to the research coordinator.

The independent variables were group allocation; socio demographic characteristics (sex, age, level of education, and marital status); and co-morbidities.

### Patient recruitment, follow-up, and adherence to the intervention

To assess the patient recruitment process, study acceptance, and adherence to the intervention, the numbers of patients who were initially contacted, who agreed to participate, who were lost to follow-up, and who received a final evaluation were recorded.

### Statistical analysis

All statistical analyses were carried out on an intention-to-treat basis using SPSS v.23. To assess the effect of CBT-i at 3 months in the measurement of quality of sleep, HADS depression score, HADS anxiety score, and changes in hypnotics and antidepressant use, between groups chi square tests were used with a confidence level of 95%. As the sample size was very small, the likelihood ratio test for contingency tables was used for comparisons where the expected frequencies were less than 5. Confidence intervals at 95% were also calculated.

This study received approval from the Majorca Primary Care Research Committee (Nº PI14-19).

## Results

### Acceptability and assessment of the training

PCPs assessed the training they received regarding the intervention during a foucs group conducted by ME. This group consisted of 12 doctors (6 in the IG and 6 in the UCG), 3 nurses (2 in the IG and 1 in the UCG). Also, the 2 psychologists participated in the group discussion. This group’s assessment of the training had several general conclusions:

- The training sessions should be longer, more practical advice should be given, and there should be more discussion of different cases.
- There is a need to provide a theoretical context for CBT-i.
- The different components of the therapy (sleep diary, identification of sleep problems, stimulus control, and relaxation) need more complete coverage.
- There is a need for more training on how to teach patients to restructure their thoughts and develop the ability to achieve concrete goals.
- The case reports described during the training sessions generated extensive input, and was considered important in clarification of some key concepts.

### Acceptability and assessment of the intervention

Regarding the recruitment of patients, both groups highlighted the difficulty in identification of eligible patients with chronic insomnia, and that most patients with insomnia symptoms had other mental disorders. GPs and nurses suggested use of no age limit, increasing the ISI cut-off to 14, and consideration of clinical assessments (i.e., poor quality of life). Also, participants in the nominal groups suggested that apart from personal invitations by PCPs, invitation posters should be installed throughout the health center and in the community. All professionals agreed that time constraints were a problem for some patients. Table 2 summarizes the positive and negative aspects of the intervention in the nominal groups.

**Table 2:** Positive aspects and difficulties of the intervention that were reported in both nominal groups or only in one nominal group

Both groups	One group only
**Positive aspects**	
• PCPs provided valuable non-pharmacological treatment • There were opportunities to go deeper into the causes of insomnia • Other sociological and sleep hygiene problems were identified • The patient-therapist relationship improved	• There was positive support for treatment of insomnia • The relaxation sessions had high value • The “Manual of Interventions” was very helpful
**Difficulties**	
• Doctors considered the goals of the intervention as too ambitious • It required PCPs to change their roles, in that it they had to address emotional issues • The intervention did not permit deep examination of other problems that emerged during the sessions • There were too many sessions, and some patients withdrew for this reason • The sessions were too short • Some concepts were repeated in the different sessions • A reorganization of agendas is needed to continue the intervention • The tutorial material for PCPs was considered essential to structure the therapy, but was too dense. More simplified materials are needed • Written material for the patient about sleep hygiene, and control of stimuli and thoughts is needed	• More time and energy are required than a normal consultation. Therapy should be given when the therapist is less tired • There was a need for preparations prior to the consultations • There were many difficulties in the session on cognitive restructuring (Session 4) • Simultaneous intervention and data collection was difficult • The intervention was more feasible for nurses, because they have more time for consultation • Patients were reluctant to work on a health problem if there is no immediate solution • Patients who had little education had difficulties completing the sleep diary

### Assessment of the recruitment process and study acceptance

**Fig. 1:**
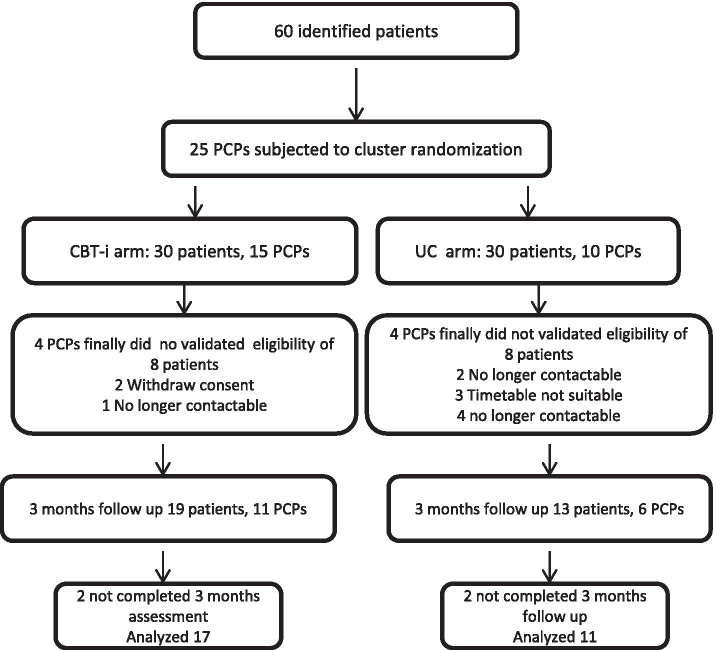
PCP participants and recruitment of patients (CONSORT flow diagram)

Figure 1 summarizes our assessment of the recruitment process and study acceptance by PCPs and patients. After study presentation in the two health centers, 25 PCPs agreed to participate (15 in the IG and 10 in the UCG). Four PCPs in each group did not recruit any eligible patients. Thus, 32 patients were recruited, 19 in the IG and 13 in the UCG. Two patients were lost to follow-up in the IG and 2 were lost to follow-up in the UCG.

### Effect of the intervention

Table 3 shows the socio demographic characteristics of the IG and UCG groups of patients. The most notable differences were that the IG had fewer married patients and fewer patients with university level education.

**Table 3:** Baseline sociodemographic characteristics and ISI scores in the intervention group (*n* = 19) and control group (*n* = 13)

**Variable**	**Intervention group** **N (%)**	**Control Group** **N (%)**
**Sex**		
Male	4 (21.1)	2 (15.4)
Female	15 (78.9)	11 (84.6)
**Marital status**		
Single/separated/divorced/widower	6 (31.6)	2 (154)
Married/couple	13 (68.4)	11 (84.6)
**Level of education**		
No secondary school	2 (10.5)	3 (23.1)
Secondary school	6 (31.6)	6 (45.2)
University	11 (57.9)	4 (30.8)
**Job status**		
Employed	11 (57.9)	4 (30.8)
Unemployed	8 (42.1)	9 (69.2)
**Insomnia grade (ISI score)**		
Subclinical insomnia (8–14)	2 (10.5)	0 (0.0)
Moderate insomnia (15–21)	14 (73.4)	11 (84.6)
Severe insomnia (≥ 22)	3 (15.8)	2 (15.4)

Table 4 shows the effects of the intervention in the two groups. The proportion of patients with short sleep latency (0–4 min) was higher in the IG (82.4%, 95%CI 56.5–96.2) than in the UCG (45.5%, 95%CI 16.7%-76.6%). In addition, higher proportions of patients in the IG group slept for longer than 5 h, 68.8% (95%CI 41.3–88.9) vs. 11.1% (95%CI 0.2%-48.2%) and had fewer sleep disruptions 88.2% (95%CI 63.5–98.5) vs. 54.5% (95%CI 23.4–83.2). Due to small numbers in each group, these differences could not be taken into account to draw any conclusions. No differences between groups were found for depression, anxiety or hypnotic use or changes in hypnotic and antidepressant drugs.

**Table 4:** PSQI results, HADS results, and use of hypnotics and anti-depressants after 3 months in the intervention group (*n* = 19) and the control group (*n* = 13)

**Variable**	**Intervention group** **n (%)**	**Control Group** **n (%)**	***P* value**
**Subjective sleep quality**			
Very good/quite good	10 (62.5)	3 (30.0)	0.107
Very bad/quite bad	6 (37.5)	7( 70.0)	
**Sleep latency (min)**			
0–4	14 (82.4)	5 (45.5)	0.041
5–6	3 (17.6)	6 (54.5)	
**Sleep duration (h)**			
5 h or more	11 (68.8)	1 (11.1)	0.006
< 5 h	5 (31.3)	8 (88.9)	
**Sleep disruptions**			
1–9	15 (88.2)	6 (54.5)	0.044
10–27	2 (11.8)	5 (45.5)	
**Use of a hypnotic**^**a**^			
None in the last month	10 (58.8)	4 (36.4)	0.408
1 or 2 times per week	2 (11.8)	1 (9.1)	
3 or more times per week	5 (29.4)	6 (54.5)	
**Day dysfunction**			
0	5 (29.4)	4 (36.4)	0.249
1–2	8 (47.1)	2 (18.2)	
3–6	4 (23.5)	5 (45.5)	
**HADS depression**^**a**^			
None (0–7)	14 (82.4)	9 (81.8)	0.971
Possible (≥ 8)	3 (17.6)	2 (18.3)	
**HADS anxiety**			
None (0–7)	9 (52.9)	6 (54.5)	0.934
Possible (≥ 8)	8 (47.1)	5 (45.5)	
**Hypnotic use**^**a**^			
No	6 (42.9)	2 (28.6)	0.276
Yes	6 (42.9)	5 (42.9)	
< 4 doses/months	2 (14.3)	0 (0.0)	
**Beginning hypnotic use**^**a**^			
Yes	0 (0.0)	1 (33.3)	0.117
No	6 (100.0)	2 (66.7)	
**Beginning antidepressant use**^**a**^			
Yes	1 (7.1)	3 (33.3)	0.107
No	13 (92.9)	6 (66.7)	

^a^ Likelihood ratio test for contingency tables

### Assessing the adherence to intervention

Five of the 19 patients in the IG did not complete all the scheduled sessions (1 attended no sessions, 1 attended one session, 2 attended two sessions, and 1 attended 3 sessions). The other 14 attended the entire schedule of 5 sessions, with a benzodiazepine withdrawal session if needed.

## Discussion

Pilot and feasibility studies play an invaluable part in health research. The results of pilot studies allow the research team to reconsider the procedures and design of the subsequent main study. The present study provided us with important information about several fundamental issues that must be addressed before beginning a large trial to assess the effectiveness of a CBT-i intervention implemented by PC doctors and nurses in patients with persistent insomnia [28, 40, 41]. The following changes are suggested.

### Intervention

The present study provided 5 sessions of CBT-i and an extra session for benzodiazepine withdrawal (if necessary). We evaluated the acceptability of this intervention using the nominal group technique to obtain opinions from the PCPs. They considered the format of the intervention as adequate, but PCPs and patients advocated the use of fewer sessions and to redesign the content of the CBT-i, by removing cognitive restructuring component because PCPs do not feel comfortable with this aspect of the intervention. Also, PCPs advocated the use of an “Intervention Guide” for PCPs as a key element, and also providing written materials for patients. They suggested that the materials for patients should cover topics such as instructions for keeping a sleep diary and a guideline for sleep hygiene, stimulus control, and control of thoughts that contribute to insomnia, and that these materials should be adapted for patients with little education. The PCPs also recommended implementation of some changes in the intervention. In particular, they considered it crucial for PCPs to make certain preparations prior to each CBT-i session, establish a specific agenda, and schedule sessions at times that are less tiring for the PCP and also consider patient availability.

### Training in CBT-i

We also identified the need to improve the training of PCPs by use of a longer training program and providing more presentations of cases that experience chronic insomnia. Thus, in the subsequent large trial, we will include some PCP suggestions about certain aspects of the training, such as to provide a more thorough discussion of the different components of therapy, to negotiate with patients the objectives of each session, and to instigate a more active follow-up of PCPs by use of periodic phone calls.

### Results of the intervention

Although this study was not designed to test a hypothesis, it showed improvements in three dimensions of the PQSI. That is, IG patients appeared to benefit from the intervention in terms of decreased sleep latency, increased sleep duration, and decreased sleep disruptions. However, we cannot place great weight on these results, because no formal sample calculations were performed and there may have been imbalances in the pre-randomization covariates. Moreover, this was an external pilot study, and the patients recruited in this work will not be included in the subsequent large study [27].

### Recruitment and follow-up of participants and patients

We had difficulties in getting doctors and nurses to participate in this trial, and some of the participants did not include any patients. In our subsequent large study, it will be necessary to work with PCPs in the recruitment of patients and implementation of the intervention. Not all PCPs have the skills to learn the basics of CBT-i, so a more careful selection of doctors and nurses should be performed in the subsequent trial.

Additionally, we found that the recruitment of patients was difficult. We therefore plan to improve the recruitment process by use of poster invitations in health centers and in the community. Furthermore, as the PCPs proposed, we will adjust the eligibility for participation in subsequent large trial. To increase patient adherence to treatment, patients will be included if they present with more severe insomnia (ISI > 14) and have poorer sleep quality. The use of more stringent criteria could increase patient motivation to participate and these patients also have greater potential for improvement. Also, reminders to PCPs and patients will be used to increase the participation.

Pilot and feasibility studies play an invaluable part of health research. We tried to be explicit as possible about the purpose of this pilot study, as proposed by Lancaster and by Arain [40, 42]. Methodological rigor is achieved by using a well-conceived and well-designed study that has clear aims and objectives, and uses a high quality definitive randomized and controlled design.

### Limitations

One of the main limitations of the study was the lack of patient involvement in the research process. We originally planned to assess patients’ opinions about the intervention, but the study ended during the summer and the residents left to work in hospitals or other health centers and could not perform this task. Fortunately, the participating PCPs reported feedback from some their patients’ comments during the nominal groups, and we will consider these comments prior to the subsequent large trial.

This study had some characteristics of a feasibility study, in that we used focus group and nominal group techniques to determine if a subsequent large trial would be feasible and reproducible. On the other hand, this study also had some characteristics of a pilot study, in that we focused on the processes to be used in a large RCT, such as recruitment, randomization, treatment, and follow-up, to identify problems that might occur in the subsequent large study.

## Conclusions

This external pilot-feasibility study indicated the need to address several key issues before performing the subsequent large clinical trial. Additional and longer training of PCPs is needed to ensure that they have sufficient skills to implement CBT-i intervention. In particular, PCPs should receive more training in cognitive therapy and increase the number of case presentations in order to gain skills in managing different possible situations. Also, in the large study, higher numbers of patients with more severe insomnia and poor quality of sleep should be invited to participate, and greater flexibility should be used to fit with the times they can attend CBT-i sessions. In addition, this preliminary work highlighted the need to adapt PCPs’ work schedules to deliver the intervention with longer and previously prepared sessions. The reporting of these practical issues in this pilot study will help to prevent similar pitfalls and mistakes in the subsequent large trial.

## Data Availability

The datasets used and/or analyzed during the current study are available from the corresponding author on reasonable request. PCPs and patients material guides for CBT-i are available in the following links: https://zenodo.org/record/4629880#.YFsyXVVKiUk and https://zenodo.org/record/4629814#.YFswu1VKiUk.

## Abbreviations

CBT-i: Cognitive behavioral therapy for chronic insomnia
IG: Intervention group
CG: Control group
HADS: Hospital anxiety and depression scale
ISI: Insomnia Severity Index
MME: Mini Mental State Evaluation
PC: Primary care
PCPs: Primary care professionals
PSQI: Pittsburg Sleep Quality Index
UC: Usual care
UCG: Usual care group
